# Supplementary figures and images for: Shell colour luminance of Cuban painted snails, Polymita picta and Polymita muscarum (Gastropoda: Cepolidae)
Source: PLoS One. 2025 Jan 15;20(1):e0314008. doi: 10.1371/journal.pone.0314008 (PMC11734995; doi:10.1371/journal.pone.0314008)

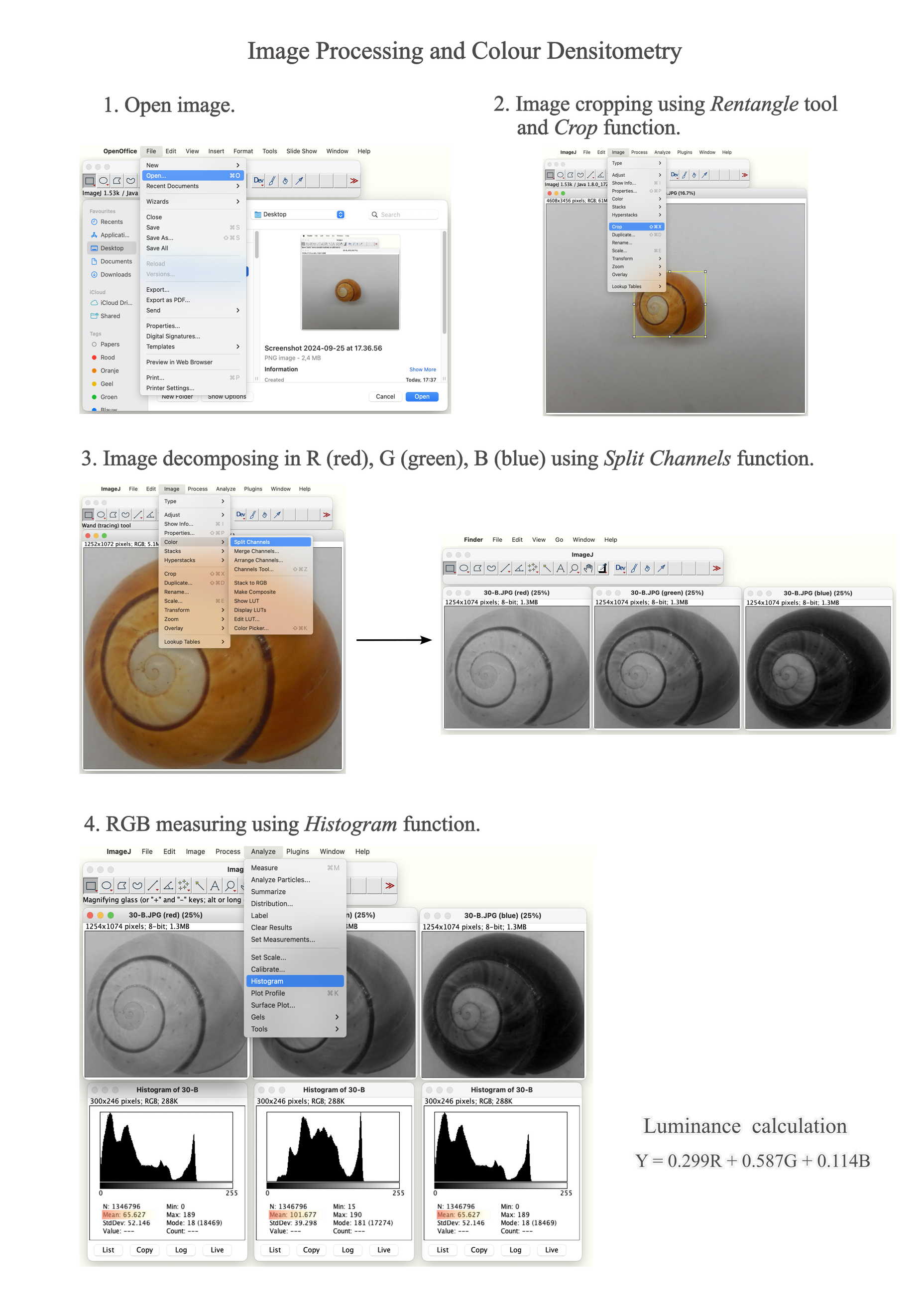

Supplement: S1 Fig — (TIF) [file pone.0314008.s005.tif]
